# Developing and Validating an Inclusive and Cost-Effective Prediction Algorithm for Survival and Death Among People Living With HIV in Sub-Saharan Africa: Protocol for a Meta-Analysis and Case-Control and Cost-Effectiveness Study

**DOI:** 10.2196/63783

**Published:** 2025-08-29

**Authors:** Martins Nweke, Julian David Pillay, Alfred Musekiwa, Sam Chidi Ibeneme

**Affiliations:** 1 Global Health and Sustainability, Faculty of Health Sciences, Durban University of Technology Durban South Africa; 2 Department of Physiotherapy, Faculty of Healthcre Sciences, University of Pretoria Pretoria South Africa; 3 School of Public Health and Health System, University of Pretoria Pretoria South Africa; 4 Faculty of Health Sciences, David Umahi Federal University of Health Sciences Uburu Nigeria

**Keywords:** HIV, survival, death, mortality, sub-Saharan Africa, prognosis, prediction

## Abstract

**Background:**

Premature death in people with HIV in sub-Saharan Africa (SSA) is highly preventable. However, the lack of inclusive, cost-effective prognostic tools remains challenging. Most prognostic tools have been developed in high-income economies. The distinct cultural dynamics in HIV-related death epidemiology makes them unsuitable for the region. Additionally, the models lack systematic stratification of death determinants based on clinical relevance, and some included factors are too expensive for people with HIV in SSA.

**Objective:**

We aimed to create a tailored predictive model that considers the unique context of SSA, including cultural dynamics, cost-effectiveness, and clinical relevance.

**Methods:**

This is a 2-phase study. In the development phase, we will use a combination of evidence synthesis, namely meta-analysis, application epidemiology, biostatistical, and economic paradigms, to develop a prognostic model for people living with HIV in SSA. The Preferred Reporting Items for Systematic Reviews and Meta-analyses (PRISMA) protocol will be followed in the structuring of the meta-analysis. From their creation to the present, we will search African journals (Sabinet) and the PubMed, Scopus, MEDLINE, Academic Search Complete, Directory of Open Access Repository, Cochrane Library, Web of Science, EMBASE, and Cumulative Index for Nursing and Allied Health Literature databases. Only cohort studies with moderate to high quality will be included. The primary outcome variables include the predictors of HIV-related death and their corresponding effect sizes (adjusted relative risk). A random-effect meta-analysis model will be used to synthesize the unbiased estimate of risk (relative risk) per predictor. Epidemiological metrics such as risk responsiveness, geotemporal trend, risk weight (Rw), clinical minimum important difference (CMID), predictors interaction density (PID), critical risk points, and potential cost implication will be computed. A combination of Rw and CMID will be used for risk stratification. The model’s constituent items will be selected based on the combination of Rw, CMID, PID and cost implication. In the validation phase, we will apply the emergent model to classify participants using a secondary data obtained from a cohort of people living with HIV in East and West Africa, with outcomes including sensitivity, specificity, calibration, and area under the receiver operating characteristic curve (AUC).

**Results:**

The study is projected to commence in October 2025 and end in September 2026. The expected result will be published in November 2026. The result will be presented using narrative and quantitative synthesis. Indices of causality namely as strength of association, temporality, consistency, biological gradient, and specificity of the predictor-outcome association will be presented in a tabular format. TheAUC will be used to decide the optimal critical risk point for the emergent predictive algorithm.

**Conclusions:**

Effective prognostication coupled with intense monitoring and evaluation, and prioritizing of therapeutic targets could positively turn around the fate of millions of people living with HIV at risk of premature death in SSA.

**Trial Registration:**

PROSPERO CRD42023430437; https://www.crd.york.ac.uk/PROSPERO/view/CRD42023430437

**International Registered Report Identifier (IRRID):**

PRR1-10.2196/63783

## Introduction

### Background

The prevalence of HIV-related death in sub-Saharan Africa (SSA) constitutes a critical public health concern, reflecting the region’s disproportionate burden within the global HIV epidemic landscape [1]. With approximately 25.6 million people living with HIV in this region, the toll of AIDS-related illnesses is starkly evident, as evidenced by the staggering figure of about 380,000 deaths in 2022 [1]. Beyond mere mortality statistics, the impact of HIV reverberates throughout families, communities, and economies, engendering multifaceted challenges and implications [2,3]. Of particular concern are adolescent girls and young women, who bear a disproportionate share of new HIV infections in the 15-to-24-year age group, accounting for over 77% of new cases in SSA [4,5]. The death of young lives because of HIV in SSA portends a negative demographic dividend for the region in the coming days [6]. Addressing this vulnerability in this demographic cohort assumes paramount importance in curbing the further spread of the virus and mitigating its deleterious consequences including premature death [7]. The high prevalence of HIV-related death not only strains health care systems but also exacerbates existing challenges within social support networks and impedes economic development efforts in the region [6]. The limited emphasis on improving survival and preventing premature death through an effective surveillance and monitoring system might be responsible for the huge burden of HIV-related death in SSA. The poor prognosis of people living with HIV in the region [7] underscores the need for a tool to rapidly and accurately predict the risk of HIV-related death in the region.

The development of an accurate and cost-effective prediction algorithm tailored specifically to the nuances of the SSA context emerges as an imperative. Such models hold the potential to not only improve survival rates but also optimize the allocation of scarce resources, thereby maximizing the impact of interventions and bolstering overall health outcomes [8]. There exist some prognostic tools for the assessment of survival or risk of death in people living with HIV. They include the prognostic nomogram by Jiang et al [9], prognostic model by Wang et al [10], shared random-effect model [11], joint latent class model [11], and the model by Auld et al [12]. However, the existing prognostic models designed to predict survival and death outcomes among individuals living with HIV are fraught with limitations vis-à-vis the SSA context, thereby necessitating the development of a new model tailored specifically for the region. Principally, these limitations encompass 4 areas including a lack of African data, ethnotemporal dynamics, statistical versus clinical significance, and heterogeneity of the African population. A lack of representation of African data within the existing models is noted as available models predominantly originate from economically affluent regions [9-11], with the exception of one model [12]. Consequently, the generalizability and applicability of these models to SSA without formal validation studies within the SSA context is questionable. Moreover, ethnotemporal dynamics, characterized by variations in the epidemiology of HIV and HIV-related mortality across different regions, pose a significant challenge to the use of existing models in the SSA. For example, respiratory disease, non-AIDS nonhepatitis malignancy, and central nervous system–related deaths were reportedly the 3 most frequent causes of death among people living with HIV in Europe, while substance abuse, respiratory disease, and non-AIDS nonhepatitis malignancy were the 3 most common causes of HIV-related deaths in North America [13]. In low- and middle-income economies, particularly the SSA, AIDS and tuberculosis were the leading causes of death in people living with HIV before 2008 and between 2008 and 2018, respectively [1,14]. These dynamics manifest through variations in cultural norms, risk factors, social determinants of health, and disease progression patterns, underscoring the need for models that are sensitive to the unique contextual factors prevalent in SSA.

Furthermore, the cost-ineffectiveness of existing models presents a formidable barrier to their use within resource-constrained settings such as SSA [15]. Incorporating factors with high investigative costs renders these models unaffordable and impractical for health care practitioners and people living with HIV in SSA. In addition, the discrepancy between statistical and clinical significance in selection of model items, otherwise known as predictors, within existing models further exacerbates their limitations [9-12]. Models prioritize predictors solely based on statistical significance risk overlooking factors that hold clinical relevance, thereby compromising the accuracy and use of the predictive model. For example, in the prognostic model by Wang et al [10], a weight of 0.5 was assigned to tuberculosis and a score of 0.6 was assigned to hepatitis C virus (HCV). From a clinical and contextual perspective, we argue that tuberculosis and HCV could not share similar risk weight (Rw) in the SSA context, where tuberculosis is the leading cause of death. The inherent heterogeneity within the African population further underscores the inadequacy of existing models, necessitating the development of models that can effectively account for and accommodate this diversity within SSA. The African race constitutes the world’s largest population of the Black race and is heterogeneous in nature [16,17]. In 2009, a genetic clustering study that genotyped 1327 polymorphism markers in various African populations identified 14 ancestral groups that share cultural or linguistic orientations and correlate with self-described ethnicity [16,18]. An Afrocentric prognostic model should be derived from the data representative of most parts of the region, while taking into consideration any possible regional and periodical variations in the determinants of HIV-related death [19].

The imperative for developing a new predictive model specific to SSA is underscored by several compelling reasons. First, such a model would be instrumental in addressing the unique cultural, epidemiological, and economic nuances prevalent in SSA, thereby enhancing its contextual relevance and applicability. Second, a tailored predictive model holds the promise of improving the accuracy of predicting HIV-related mortality within the region, thereby facilitating more informed clinical decision-making and resource allocation. Finally, the development of a cost-effective and clinically relevant predictive model holds immense potential in equipping health care practitioners in SSA with the necessary tools to effectively manage and mitigate the impact of HIV within their respective communities [20,21].

### Research Question

Can an inclusive and cost-effective predictive model be developed and validated for predicting survival and death in people living with HIV in SSA?

### Aim

We aimed to create a tailored predictive model that considers the unique context of SSA, including cultural dynamics, cost-effectiveness, and clinical relevance. By validating this model using real-world data, researchers seek to enhance accuracy in predicting HIV-related outcomes and improve health care strategies for people living with HIV in the region.

## Methods

In this study, we will use a 2-phase approach. The methodology for each phase is presented in subsequent sections.

### Phase 1

The aim is to develop an inclusive and cost-effective predictive model for survival and death in SSA. We will use a novel technique involving meta-analysis, epidemiological, biostatistical, and economic paradigms to assemble the emergent model. This will address the shortcomings of the current predictive or prognostic models, which include the glaring omission of African data, the inability to resolve the enormous disparity between statistical and clinical significance and ethnotemporal dynamics in the epidemiology of HIV-related death, and cost-ineffectiveness.

#### Population, Exposure, Comparison, Outcome, and Time Criteria

The population of the review includes a cohort of people living with HIV with known exposure status who were followed up until the incidence of deaths, as in a prospective cohort; individuals who were reported dead as a result of HIV and who were retrospectively followed up to identify the cause and determinant of death, as in a retrospective cohort [22]. The primary outcomes are determinants of survival in people living with HIV such as age, route of HIV acquisition, CD4 count, coinfection with tuberculosis, coinfection with HCV, obesity (BMI), hemoglobin, CD4 cell count, platelet count, aspartate transaminase, income, aloneness, and plasma glucose, among others. The corresponding risk estimate (adjusted relative risk) for each predictor. In addition, outcomes such as risk estimates namely risk responsiveness (Ri), predictors interaction density (PID), geotemporal trend, clinical minimum important difference (CMID), and risk weight (Rw) Rw will be derived [19,23,24]. The review will cover the time between the inception of the oldest database (1961) and May 2025.

#### Protocol and Registration

This protocol is registered with the International Prospective Register of Systematic Reviews (PROSPERO; CRD42023430437). A systematic review using a meta-analysis technique, risk stratification, and ethnotemporal analysis of homogeneity of the predictors of HIV-related death in SSA will be used to generate the model items. The systematic review component will be structured using the Preferred Reporting Items for Systematic Reviews and Meta-analyses (PRISMA) checklist [25] and adhered to the recommendations of Meta-analysis of Observational Studies in Epidemiology [26].

#### Eligibility Criteria

The inclusion and exclusion criteria for the studies are presented in Textbox 1.

**Inclusion and exclusion criteria for studies.**

**Inclusion criteria**

- Studies must be peer-reviewed sub-Saharan African (SSA) studies.
- Studies must use reported predictors or determinants of HIV-related death in SSA and their corresponding risk estimates (relative risk [RR] or hazard ratio) or provide sufficient information for the risk estimate to be calculated and transformed into RRs.
- Studies that used a cohort study design.
- Studies with low or moderate risk of bias.
- A predictor has to be reported by at least 3 studies to be included in the meta-analysis. There will be no restriction regarding study setting, period of study, age, and gender. However, only studies conducted among adults will be included.

**Exclusion criteria**

- Cross-sectional studies.
- Studies that have a high risk of bias.
- Studies that have been published in languages other than English or French.
- Studies that have no reported risk estimate (ie, RR or its equivalent), despite reporting predictors of HIV-related death.

#### Sources of Information

We will search the MEDLINE**,** PubMed, Academic Search Complete, EMBASE, Cochrane Library, CINAHL, Web of Science, and Scopus databases and African journals (Sabinet) from inception (1966) to January 2025. In addition, we will search the Directory of Open Access Repository. A piloted and refined PubMed search strategy is provided in Multimedia Appendix 1. The long list of the databases is to ensure that no eligible articles are left out.

#### Search Strategy

An information specialist, together with MN developed, tested, and refined the search strategy. Search terms were identified from the key concepts in the review title. A variety of terms from medical subject categories were used in each search. To obtain comprehensive results, a combination of index terms, keywords, and Boolean operators (AND and OR) was used. To determine whether the search strings were appropriate, a pilot PubMed search was conducted (Multimedia Appendix 1). Keywords were searched in the title, abstract, and keyword fields, in addition to MeSH terms. We will also use truncation and wildcards for the keywords. The search terms will be modified to match the subject headings and syntax of the remaining databases: MEDLINE, PubMed, EMBASE, Academic Search Complete, Cochrane Library, CINAHL, Web of Science, Scopus, and Directory of Open Access Repository and African journals (Sabinet).

#### Data Management

Duplicate entries will be removed from the literature search results after they are exported to EndNote 20 (Clarivate). Following the removal of duplicates, the articles will undergo a preliminary title and abstract screening using EndNote 20. The full texts of all eligible articles will be reviewed for potential exclusion. EndNote 20 will also be used to organize and export included and excluded articles for generating the PRISMA flowchart and in-text citations.

#### Study Selection and Data Extraction

The initial screening of titles and abstracts will be conducted by 2 trained research assistants: Kenechukwu Maryjane Ukwuoma and Praise Peter-Oyirinnaya. The principal investigator, MN, will undertake the training of the research assistants. In the event of conflicting results, discussion between Kenechukwu Maryjane Ukwuoma and Praise Peter-Oyirinnaya, followed by consultation with MN as an impartial reviewer, will be used to resolve discrepancies. Data extraction will be executed independently by Kenechukwu Maryjane Ukwuoma and Praise Peter-Oyirinnaya. Similarly, any conflicting results will be resolved through discussion and in consultation with MN, SCI, and AM. We will contact the relevant authors of the full-text articles in the event of missing relevant data items. The flow of the studies during the selection process and the justification for exclusion will be shown in detail in the PRISMA diagram.

#### Outcomes and Data Items

Outcome measures may include both laboratory and nonlaboratory measures. These may include, but are not limited to, age, route of HIV acquisition, CD4 count, coinfection with tuberculosis, coinfection HCV, obesity, hemoglobin, CD4 cell count, platelet count, aspartate transaminase, and plasma glucose. The methods or techniques used to determine and quantify the predictors will be reported to examine their comparability in the SSA setting. Secondary data will include study characteristics such as type of HIV, stage of HIV, setting, study design, sample size, and period of study, among others

#### Risk of Bias Assessment

We will assess the risk of bias in the studies using the Joanna Briggs Institute risk of bias assessment tools for cohort studies [27,28]. The risk-of-bias assessment will be carried out by the research assistants. Before assessment, MN will train the research assistants. Several training sessions will be undertaken until an interrater agreement of at least 80% is achieved between MN and Kenechukwu Maryjane Ukwuoma and Praise Peter-Oyirinnaya each. To obtain the interrater agreement, we will calculate the ratio of the total number of articles accurately rated by MN, Kenechukwu Maryjane Ukwuoma, and Praise Peter-Oyirinnaya each to the total number of the eligible full-text articles.

#### Effect Measures

Primary basic data will include the predictors or determinants and their corresponding estimates (relative risk [RR]) or proportion of events used for the computation of RR and the risk of bias score. The derived primary data items will include the responsiveness (Ri), CMID, Rw, and critical risk point (CRP).

#### Grading of Evidence

The strength of the evidence on the predictive potential of the determinants of death among people living with HIV in SSA will be evaluated using the Grades of Recommendation, Assessment, Development, and Evaluation (GRADE) [29] as presented in subsequent sections:

#### Bias Risk

The Joanna Briggs Institute risk-of-bias assessment tool for cohort studies [27] will be used to evaluate the quality of the eligible articles. Risk of bias will be categorized into low, moderate, and high risk of bias. The grade of evidence otherwise known as the level or certainty of the evidence may be lowered by 1 level for high risk of bias or by 2 levels for moderate risk of bias, respectively, if there are significant concerns about the possibility of bias [29].

#### Inconsistency

This refers to significantly different effect estimates that are obtained from the variation or heterogeneity in research findings. It is indicative of the true differences in effect estimate (less bias) [30]. Inconsistency will be assessed using the *I*^2^. *I*^2^ values indicate greater inconsistency (<40% is considered low, 30%-60% is considered moderate, 50%-90% is considered substantial, and 75%-100% is considered significant) [30]. Evidence will be downgraded by one level in the event of significant heterogeneity [30].

#### Indirectness

Variations in patients, exposures, comparisons, or results may render the evidence for a research question implicit. According to Guyatt et al [31], the certainty of the evidence will be reduced by one level when there are notable variations between the population, comparisons, or outcomes of interest and those measured in the available evidence.

#### Imprecision

Research involving a small number of patients, a small number of events observed, or a large degree of variability in patient effects typically yields results with broader CIs and less precision [32]. If a study’s CI is larger than the predetermined threshold or its dataset is not sufficiently large, diminishing the evidence’s certainty, it is deemed imprecise. Succinctly, a finding may be termed imprecise if the CIs, in relation to the clinically important difference, are overlapping. As a general guideline, RRs of 0.75 and 1.25 will be used to evaluate the precision of the findings [32].

#### Publication Bias

When studies are not fully reported in the published literature, biases in reporting may arise. According to Guyatt et al [33], publication bias increases studies’ eligibility for inclusion in a systematic review or guideline. Hence, the existence of publications may reduce the grade of evidence by 1 or 2 points, depending on the severity of bias [33].

#### Risk Estimate

RR will be used in this study to evaluate a given predictor’s predictive potential, which will be obtained through meta-analysis. When there is a minimum 2-fold decrease or increase in risk of death, the quality of the evidence will be rated one level higher; when there is a minimum 5-fold reduction or increase in risk, the rating will be 2 levels higher [34].

#### Data Synthesis and Analysis

All eligible studies were included in the data synthesis. Mean and SD will be used to describe the quantitative characteristics of the participants such as age, duration of HIV, duration on antiretroviral therapy, age at death, length of follow-up, medication adherence rate, and other quantitative study characteristics such as ample size. The participants’ educational achievements, gender, type of cohort design, setting, country, period of study, and sampling technique will be summarized in percentages.

#### Meta-Analysis

First, using a prespecified evidence table, we will undertake a narrative synthesis to examine the distribution of the predictors of HIV-related death in SSA based on study setting, country, subregion, and period of study. All risk estimates (effect sizes) will be converted to RR when appropriate and for the sake of comparison [35-37]. To group all effects into a single frame, we will convert RR to 1/RR when RR <1 [38]. Similarly, we will use a multiplier effect (1/RR^2^) to frame the CI when RR <1. The pooled RR as well as measures of heterogeneity (*I*^2^) and publication bias will be calculated for each predictor using the random-effect meta-analysis model. The comprehensive meta-analysis software, which uses the inverse variance method, will be used to synthesize the aggregate RR for each predictor. The CRP will then, correspondingly, be found in the fourth quartile. Statistical analysis will be supplemented with the SPSS (IBM Corporation). The level of significance was set at Cronbach α=0.05.

#### Meta-Bias

To assess publication bias, we will generate and inspect a funnel plot, with its symmetry tested using the Egger test. Publication bias will be considered present when a statistical significance demonstrating the asymmetry of the funnel plot is found [39].

#### Confidence in Cumulative Evidence

To assess the confidence in the quality of evidence used to construct the model, we will use the GRADE as previously described [40]. The GRADE assigns 4 levels of confidence, certainty, or grades—namely high, moderate, low, or very low—depending on study quality, precision, consistency, directness, and absence of publication bias and size of effect estimate, which in our context is the RR [41].

#### Determining the Clinical Responsiveness and Risk Weight of the Identified Predictors

The proportion of the studies reporting clinical significance for a particular predictor is referred to as risk responsiveness or risk stability (Ri), provided there is no publication bias. The Ri is given by f_s_/∑f. It is a measure of how consistently a given factor is said to be a predictor, provided there is no publication bias otherwise known as selective reporting. Rw is a multiplicative function of Ri and risk estimate (RR). Hence, Rw=RR×Ri [19].

#### Examining the Homogeneity or Inclusiveness of Predictors of HIV-Related Death in SSA

A predictor will be said to be homogeneous (Afrocentric) if it is reported and found to be clinically relevant in at least 3 of the 4 traditional SSA regions, with a geotemporal trend of at least 0.6. However, we provide a narrow window of exceptions for emerging risk factors for HIV-related mortality that are associated with high investigative cost. In total, we will guarantee that 3 out of 4 criteria are met by 75% of the predictors that will be incorporated into the predictive model [19].

#### Stratification of the Predictors of HIV-Related Death Using Emerging Indexes of Clinical Relevance

Stratification of the predictors of HIV-related deaths in SSA will be carried out using Rw, which is a product function of the risk estimate (RR) and Ri. As long as there is no publication bias or selective reporting, the Ri is a measure of how consistently a given factor predicts the death. It will be computed as follows: Ri=fs/∑f, where fs is the number of publications or articles classifying a given predictor of HIV-related death as a clinically relevant factor and ∑f is the total number of studies that reported a given predictor of HIV-related death in SSA [19].

#### Determining the CRP

The CRP is the point on a risk curve at which risk of the outcome under consideration is incident and where, in a high-risk preventive strategy, a pre-emptive intervention should be commenced [19]. The CRP falls in the upper risk quartile (between the 76th and 100th percentile) of the Rw distribution for high-risk strategies or targeted screening. These points should also be precisely high enough to include most clinically significant markers. Using quartile division, the generated risk point will be divided into 4 classes. The Rw values in the lower, middle, and upper classes correspond to first (25th percentile) and second quartile (≥50%) and third quartile (51%-≥75th percentile) [19].

#### Selection of Model Components or Items

The CMID, which is defined as a change in RR by at least 50% [32], will be combined with the Rw to determine which model components (predictors) to include [19]. The CRP will be considered during the selection process. While there are multiple ways to determine risk based on the CRP, factors that are cost-effective, clinically relevant, and inclusive will be given priority [19].

#### Determining Cost-Effectiveness

A transaction is considered cost-effective when maximum benefit is obtained at a relatively small cost [42]. Any prudent combination of responsive and clinically relevant predictors or biomarkers can be used to build the model by computing the CRP, as long as the combination satisfies the critical point [19]. In this study, prudence will be defined as high model performance and cost-effectiveness. The average investigative cost associated with these responsive and clinically relevant model factors or biomarkers, as obtained from the representative SSA nations, will serve as the basis for defining cost-effectiveness.

Laboratories in the representative nations will provide information on the investigative cost associated with identifying and measuring the clinically relevant predictors. According to Nweke et al [23], a representative country in a given region (Central, East, Southern, or West Africa) is one that has contributed the most data toward the development of the HIV-related death predictor in that region. We will use the dollar-to-currency midrate histories [43] to make the necessary adjustments in inflation rate gaps where price data were obtained from a year before 2025. The formula for calculating the inflation rate is (B−A)/A×100, where A represents the midrate from the year before and B represents the rate from the current year. Therefore, when going from the year before to the current year, the new adjusted price is equal to (Old price+[inflate rate×B]); when going the other way, it is equal to (New price−[inflate rate×B]) [19]. Prices will be gathered and converted into US dollars for comparison’s convenience. If the associated investigative cost falls within the average cost, the predictor—also referred to as a model component—will be considered cost-effective. According to Nweke et al [19], a model is considered cost-effective if the expenses associated with the investigation are comparable to the average cost.

To determine the cost-effectiveness of the emerging model, the components of the emergent model and those of comparator models—prognostic model by Wang et al [9], model by Jiang et al [10], and model by Auld et al [12]—will be listed in the order of increasing cost. Next, we will choose a specific risk level or category, such as low, moderate, or high risk as defined in the comparators, and investigate every avenue that could lead to the risk category in the emergent model. We will then calculate the average costs for the various paths taken to reach the specific risk level in both emergent and comparator models. Finally, the computed average cost implication of using the emergent model will be compared with those of the comparator prognostic models to determine the cost benefit of using the emergent model.

Using the summative Rw, we will analyze each model’s overall ability to predict HIV-related death to evaluate the efficacy (performance) of the emerging model. If the summative or cumulative Rw of a model is at least equal to the CRP, the model will be deemed effective. Therefore, a model will be termed cost-effective if is “less costly and at least as effective or more effective and costlier, with the added benefit worth the added cost, or less effective and less costly, with the added benefit of the alternative not worth the added cost or cost saving with an equal or better outcome” [44]. Interestingly, any logical combination of responsive and clinically relevant predictor or biomarkers may be used to create the model as long as the CRP is reached [23].

To test the hypothesis that the emerging model will be more cost-effective than the comparators, we will define cost-effectiveness in terms of the incremental or marginal cost-effectiveness ratio [45]. In this context, it refers to the cost of using the emerging model instead of the prognostic model by Wang et al [10]. Incremental cost-effectiveness is defined as the ratio of the difference in cost to the difference in effectiveness, that is, the cost difference between the emergent model and each comparator divided by the corresponding difference in performance as measured by the Rw [45].

### Phase 2

Phase 2 is the validation of the inclusive and cost-effective predictive model for survival or death in SSA, using a secondary data analysis of a prospective Arican cohort.

#### Design

This is the secondary analysis of data from a prospective African cohort of people living with HIV. The study aims to validate an emergent predictive model for survival or death among people living with HIV in SSA. Hence, the study will be conducted as per the guidelines by Cuschieri [46] and supplemented by the Standardized Reporting of Secondary Data Analyses–2 checklist [47]. In this study, we will apply the emergent model to a secondary dataset to test its performance.

#### The Emergent Model

The emergent predictive model for survival or death among people living with HIV tailored for SSA will mark a significant advancement in addressing the unique challenges surrounding HIV-related survival and mortality in the region. Unlike existing models, this innovative approach accounts for SSA’s contextual intricacies, including cultural, epidemiological, and economic factors. It will incorporate predictors relevant to the diverse African population, ensuring inclusivity and accuracy. Moreover, it will prioritize cost-effective predictors, facilitating wider adoption within resource-constrained SSA. Emphasizing clinical relevance over mere statistical significance, the model enhances predictive accuracy by considering factors directly associated with HIV-related death in SSA. Dynamic in nature, the model adapts to evolving trends, ensuring continued relevance and effectiveness in predicting outcomes. Comprehensive in scope, it will cover a wide range of predictors, capturing the multifaceted nature of HIV-related outcomes in SSA. Therefore, this emergent predictive model will offer a culturally sensitive, cost-effective, and clinically relevant approach to improving survival predictions for people living with HIV in SSA.

#### Unit of Analysis

The unit of analysis will comprise the dataset from Kibuuka et al [48], which examined the incidence, causes, and the predictors of death among people living with HIV. The ongoing study was established in 2013 and longitudinally assesses the impact of clinical practices, biological factors, and sociobehavioral issues on HIV infection and disease progression in an African context as previously described [49]. The study was carried out at 12 sites in selected SSA settings, namely Kenya, Uganda, Tanzania, and Nigeria. Incidence of death and predictors of death among people living with HIV constitute the primary outcomes.

#### Participants, Sampling, and Sample Size

Choosing the dataset for validation by Kibuuka et al [48] offers several compelling reasons: first, it provides real-world data on HIV-related death among people living with HIV, encompassing known properties such as variance, collinearity, and multivariate dependence. This ensures that the validation process accurately reflects the conditions encountered by health care practitioners. Second, the dataset was derived from a study conducted across 12 sites in SSA (Kenya, Uganda, Tanzania, and Nigeria), aligning closely with the context for which the emergent model is being developed. This geographic representation captures the diverse epidemiological dynamics specific to the region. Moreover, the dataset includes varying sample sizes (n=50, 500, and 5000), enabling the assessment of model performance across different data availability scenarios. Furthermore, the dataset contains predictor profiles relevant to HIV-related death, such as CD4 count, viral load, and clinical factors. These predictors resonate with the emergent model’s emphasis on clinical relevance and cost-effectiveness. Hence, the dataset by Kibuuka et al [48] presents a valuable opportunity to validate the emergent predictive model within a representative SSA context, considering both statistical properties and real-world applicability.

#### Eligibility Criteria

The inclusion and exclusion criteria for datasets are presented in Textbox 2.

**Inclusion and exclusion criteria for datasets.**

**Inclusion criteria**

- Datasets of people with HIV whether surviving or dead
- Datasets of participants aged ≥18 years
- Datasets of participants in which death was confirmed through autopsy or from a relative
- Datasets of people living with HIV whose predictor profiles were completely reported

**Exclusion criteria**

- Datasets of people living with HIV whose risk predictor profiles were incompletely reported (with missing relevant variables) will be excluded

#### Data Collection and Organization

#### Variables

In this study, our primary variables will include predictors of death in the cohort. The predictors of HIV-related death include CD4 ≤350, viral load, recent fever, BMI <18.5 kg/m^2^, clinical depression, World Health Organization stage III, hemoglobin, and age. Our secondary outcome variables include sensitivity, specificity, calibration, discrimination, the optimal risk score, and concurrent validity of the emergent predictive model.

#### Instruments

Data on the predictor profile of the participants are available in the datasets [48]. Hence, no further instrumentation will be required. Risk of deaths among people living with HIV will be calculated using the emergent Afrocentric predictive model and 4 major existing risk models, namely the model by Auld et al [12], by Wang et al [10], and by Jiang et al [9]. The diagnostic or predictive properties including sensitivity, specificity, calibration, discrimination, the optimal risk score, and concurrent validity of the emergent predictive model will be established through statistical analysis.

#### Validation Procedure

To evaluate the performance, namely sensitivity, specificity, calibration, discrimination, area under the curve (AUC) or optimal score, and concurrent validity of the emerging model, we will apply the emergent predictive model and the 4 previous models to the 2724 datasets of people living with HIV with or without death and score them individually. The diagnostic properties will be calculated for the emergent model and each of the 4 previous models. The performance of the emergent model will be compared with the 4 preexisting models.

#### Data Analysis

For the validation of the emergent predictive model, we will use specific statistical methods and analyses. These statistical methods will rigorously evaluate the emergent predictive model’s accuracy, discrimination, and calibration using the dataset by Kibuuka et al [48]. Receiver operating characteristic (ROC) analysis assesses the model’s discriminatory power by plotting the true positive rate (sensitivity) against the false positive rate (1-specificity) across different threshold values. The area under the ROC curve quantifies overall performance, with a higher AUC indicating better discrimination. Calibration and discrimination analysis evaluates how well-predicted probabilities align with observed outcomes using calibration plots or Hosmer-Lemeshow goodness-of-fit tests. Ideally, predicted probabilities should match actual outcomes across different risk groups. We will calculate the Harrel C-statistic and Somer D-statistic to measure discrimination and the H-L test to measure calibration.Sensitivity and specificity measure the proportion of correctly predicted positive outcomes (deaths) and negative outcomes (survival), respectively. These metrics will be calculated at various threshold levels to assess model performance. Optimal risk score determination involves identifying the threshold that maximizes both sensitivity and specificity, thereby balancing predictive accuracy for high-risk individuals. Concurrent validity assesses how well the emergent model aligns with existing models (eg, those by Auld et al [12], Wang et al [10], and Jiang et al [9]) by comparing performance metrics (AUC, sensitivity, and specificity) between the emergent model and comparator models.

All data analyses will be performed using R (version 3.6.2) and SPSS (version 30).

## Results

The study commenced in May 2025 and is projected to end in October 2025. The expected results will be published in November 2025. The selection process for included and excluded studies will be described and presented using the PRISMA flow diagram. The results of the individual studies will be presented using narrative and quantitative synthesis. The results of risk assessments for each eligible study will be presented in a tabular format. Indices of causality, such as strength of association, temporality, consistency, biological gradient, and specificity of the exposure outcome association, will be synthesized and presented in tables. Heterogeneity, publication bias, and sensitivity will be reported alongside the meta-analysis results in a tabular format. The emergent and cost-effective model will be represented using Rothman causal pie (set) model. The ROC will be used to decide the optimal CRP for the emergent predictive algorithm. On the basis of the CRP, a high-risk model will be fitted, taking into consideration the CMID, PID and potential cost.

## Discussion

### Anticipated Findings

We anticipate developing a highly accurate and cost-effective algorithm for predicting survival and death among people living with HIV, with the potential of being transformed into a mobile app. Of the 14 prognostic models, 13 were developed in high-income countries [49-61], 1 in China [10], and none in Africa. Hence, our proposed model will be the first prognostic algorithm among people living with HIV to be derived from African data. The conversion of the model into an app will improve accessibility and use across the region [62]. Effective prognostication coupled with intense monitoring and evaluation, and prioritizing of therapeutic targets, possesses the potential to positively transform the fate of millions of people living with HIV at risk of death in SSA [63]. Intense monitoring and evaluation of people living with HIV at risk of death in SSA could be achieved by translating the emergent model into an app that is capable of data capturing and communication. In discussing the findings of the study, diagnostic properties including sensitivity, specificity, and total accuracy will be compared across models. Existing models [49-61] exhibit varying sensitivity and specificity levels based on their predictor selection and generalizability. Existing models may prioritize specificity differently, potentially at the expense of sensitivity or vice versa. Trade-offs between sensitivity and specificity are common but should be determined on whether the predictive algorithm is a part of a population-wide strategy or a more targeted nonmass strategy [64]. Existing models may strike different balances, resulting in varied diagnostic properties. High specificity may be preferred in a high-risk (targeted) preventive strategy, while a high sensitivity and low and scalable screening may be preferred in a mass preventive strategy [64], especially for a serious and modifiable outcome [65] such as death in people living with HIV. The AUC, summarizing a model’s discriminatory power, reflects its ability to distinguish between deaths and survivals [66]. Comparing AUC values across models elucidates their relative performance in this regard. Clinically, high sensitivity aids in early detection and intervention, while high specificity ensures accurate predictions, minimizing false alarms [66]. The emergent model’s balance between these properties will impact its practical use. Therefore, comparing diagnostic properties across models provides insights into their relative strengths and weaknesses.

Should the emergent model surpass existing models in performance, clinicians stand to benefit from enhanced accuracy in risk predictions. This improvement translates directly into more effective patient care strategies, enabling targeted interventions tailored to individual patient needs. Particularly noteworthy is the potential for early identification of high-risk people living with HIV, facilitated by the model’s high sensitivity. This early detection allows for proactive monitoring and intervention, with the aim of averting adverse outcomes and improving patient prognosis. Moreover, the model’s cost-effectiveness offers health care systems in SSA an opportunity to optimize resource allocation, ensuring that interventions are prioritized based on the model’s recommendations, thus maximizing the impact of limited resources. However, to maximize the potential benefits of the proposed algorithm, the model will be applied to inform the construction of evidence-based mobile apps for the prediction of survival or death among people living with HIV. The validated app will be introduced to patients early to determine prognosis and informed treatment intensification strategies, in view of available resources. The app may be used by individuals, with the data reviewed periodically to monitor the risk of death and inform actional steps in the care for people living with HIV.

### Limitations

Although the CRP may fall within the upper quartile (76th-100th percentiles) for a targeted nonmass strategy, and 51-75th percentiles for mass strategy, the subjectivity in deciding the CRP at the development stage constitutes a limitation. However, this is simply a reflection of the imbalance between sensitivity and specificity. High CRP will definitely be more specific and less sensitive, whereas a lower CRP will be more sensitive but less specific. This dichotomy will be resolved during the validation with the ROC analysis sued to refine and identify the optimal CRP. Although, the dataset by Kibuuka et al [48] provides a list of predictor variables relevant to people living with HIV, it is possible that it does not include all putative predictors of death or survival in people living with HIV of the SSA extraction. This constitutes a limitation and might limit the number of relevant and interactive paths through which predictors may induce and facilitate survival or death in people living with HIV.

### Future Research Directions

Looking ahead, several avenues for future research emerge. Independent validation of the emergent model across diverse SSA regions is imperative to ascertain its robustness and generalizability. Longitudinal studies exploring the model’s performance over extended follow-up periods offer insights into its stability and adaptability to evolving health care contexts. Furthermore, successful validation may pave the way for the integration of the model into electronic health records or decision support systems, thereby enhancing its practical utility in clinical settings. Research efforts should also extend to investigating the impact of model predictions on patient-centered outcomes such as quality of life and survival rates, thereby elucidating the broader implications of model implementation.

### Conclusions

Effective prognostication coupled with intense monitoring and evaluation and prioritizing of therapeutic targets could positively turn around the fate of millions of people living with HIV at risk of death in SSA.

## Appendix

Multimedia Appendix 1 PubMed Search strategy.

## Abbreviations

AUC: area under the curve
CMID: clinical minimum important difference
CRP: critical risk point
GRADE: Grades of Recommendation, Assessment, Development, and Evaluation
HCV: hepatitis C virus
PRISMA: Preferred Reporting Items for Systematic Reviews and Meta-analyses
PROSPERO: International Prospective Register of Systematic Reviews
RR: relative risk
Rw: risk weight
SSA: sub-Saharan Africa

## Data Availability

The study data are available from the corresponding author upon reasonable request.
