# Supplementary material for: Developing and Validating an Inclusive and Cost-Effective Prediction Algorithm for Survival and Death Among People Living With HIV in Sub-Saharan Africa: Protocol for a Meta-Analysis and Case-Control and Cost-Effectiveness Study
Source: JMIR Res Protoc. 2025 Aug 29;14:e63783. doi: 10.2196/63783 (PMC12432474; doi:10.2196/63783)
Supplement: Multimedia Appendix 1 [file resprot_v14i1e63783_app1.docx]

Appendix 1: PubMed Search strategy

|  | Database searched | Date of Search | Filter Applied | No Retrieved |
| --- | --- | --- | --- | --- |
| (((HIV OR HIV infection OR Acquired Immune Deficiency Syndrome Virus OR Acquired Immunodeficiency Syndrome Virus OR HIV/AIDS OR HIV/AID OR AIDS OR AIDS Viruses[MeSH Terms]) AND (Death OR deaths or survival OR mortality OR mortalities OR deaths OR fatality[MeSH Terms])) AND (Predictors OR predictor OR Association* OR associated* OR correlates OR Correlated* OR determinant OR determinants OR determinant OR factors associated* OR prediction OR predictive OR predicted OR prognos*[MeSH Terms])) AND (Cohort OR Cohort analysis OR cohort study OR Cohort studies[MeSH Terms]) | Pubmed | 10/05/2024 | No filter | 14,921 |
